# Combining phenotypic and proteomic approaches to identify membrane targets in a ‘triple negative’ breast cancer cell type

**DOI:** 10.1186/1476-4598-12-11

**Published:** 2013-02-13

**Authors:** Steven Rust, Sandrine Guillard, Kris Sachsenmeier, Carl Hay, Max Davidson, Anders Karlsson, Roger Karlsson, Erin Brand, David Lowne, John Elvin, Matt Flynn, Gene Kurosawa, Robert Hollingsworth, Lutz Jermutus, Ralph Minter

**Affiliations:** 1 MedImmune, Granta Park, Cambridge, CB21 6GH, UK; 2 MedImmune, One MedImmune Way, Gaithersburg, MD, 20287, US; 3 Nanoxis AB, Lennart Torstenssonsgatan 5, SE-40016, Gothenburg, Sweden; 4 Division of Antibody Project, Institute for Comprehensive Medical Science, Fujita Health University, Toyoake, Aichi, Japan

**Keywords:** Phage display, Hybridoma, Antibody, Phenotypic screening, Membranome, CD73

## Abstract

**Background:**

The continued discovery of therapeutic antibodies, which address unmet medical needs, requires the continued discovery of tractable antibody targets. Multiple protein-level target discovery approaches are available and these can be used in combination to extensively survey relevant cell membranomes. In this study, the MDA-MB-231 cell line was selected for membranome survey as it is a ‘triple negative’ breast cancer cell line, which represents a cancer subtype that is aggressive and has few treatment options.

**Methods:**

The MDA-MB-231 breast carcinoma cell line was used to explore three membranome target discovery approaches, which were used in parallel to cross-validate the significance of identified antigens. A proteomic approach, which used membrane protein enrichment followed by protein identification by mass spectrometry, was used alongside two phenotypic antibody screening approaches. The first phenotypic screening approach was based on hybridoma technology and the second was based on phage display technology. Antibodies isolated by the phenotypic approaches were tested for cell specificity as well as internalisation and the targets identified were compared to each other as well as those identified by the proteomic approach. An anti-CD73 antibody derived from the phage display-based phenotypic approach was tested for binding to other ‘triple negative’ breast cancer cell lines and tested for tumour growth inhibitory activity in a MDA-MB-231 xenograft model.

**Results:**

All of the approaches identified multiple cell surface markers, including integrins, CD44, EGFR, CD71, galectin-3, CD73 and BCAM, some of which had been previously confirmed as being tractable to antibody therapy. In total, 40 cell surface markers were identified for further study. In addition to cell surface marker identification, the phenotypic antibody screening approaches provided reagent antibodies for target validation studies. This is illustrated using the anti-CD73 antibody, which bound other ‘triple negative’ breast cancer cell lines and produced significant tumour growth inhibitory activity in a MDA-MB-231 xenograft model.

**Conclusions:**

This study has demonstrated that multiple methods are required to successfully analyse the membranome of a desired cell type. It has also successfully demonstrated that phenotypic antibody screening provides a mechanism for rapidly discovering and evaluating antibody tractable targets, which can significantly accelerate the therapeutic discovery process.

## Background

Antibody therapies are well established as part of the treatment strategy against cancer. However, a key challenge for this therapeutic drug class is the ongoing identification of antibody tractable targets. This is illustrated by the finding that the majority of monoclonal antibodies (mAbs) approved for clinical use or in clinical development target a relatively small number of antigens; e.g. EpCAM, MUC1, EGFR, CD20, CEA and HER2 [1]. The advent of transcriptomics- and proteomics-based methods has led to the identification of a large number of candidate targets in a variety of cancer types, including breast cancer [2] and melanoma [3]. However, these techniques are not necessarily suited to the specific identification of tractable antibody targets [4] and can require significant target validation efforts to determine disease-relevant function. An alternative approach to target identification is phenotypic antibody screening, where antibodies are selected for functional activity rather than target specificity [5]. The target of each antibody is then identified later on in the process.

The success of phenotypic screening approaches in the discovery of first-in-class small molecule drugs has recently been highlighted [6]. The strength of phenotypic screening is that the focus of the approach is not constrained by preconceived target biology or molecular mechanisms of action, allowing the freedom to discover potentially novel drug targets and, for targets that are already known, novel mechanisms of action. The use of antibody technology for phenotypic target discovery has been dominated by the use of hybridoma-based techniques. However, improved antibody isolation and target identification techniques combined with the incorporation of high-throughput functional screens have led to increased success using phage display-derived antibodies [7]. The phenotypic antibody screening approach for target discovery has the advantage that the isolated antibodies can also be used for validation activities and in some instances can even be pursued as therapeutic candidates.

This study explores three different approaches to target discovery, using the MDA-MB-231 breast cancer cell line [8] as a model system. The MDA-MB-231 cell line was selected as a ‘triple negative’ breast cancer cell line, which lacks expression of the estrogen receptor (ER) and the progesterone receptor (PR) and does not amplify or overexpress HER2/ErbB2. In patients, this breast cancer subtype is aggressive and has few treatment options; hence it represents a disease type and patient population that requires new therapeutic agents [9]. Using this cell line, we evaluate the performance of two phenotypic antibody screening approaches, one based on hybridoma technology and one based on phage display technology. These phenotypic approaches are contrasted with a global proteomic survey of membrane proteins present in the cell line. Antibodies isolated by the phenotypic approaches are tested for cell specificity as well as internalisation. Screening for internalisation allows for the identification of targets potentially useful for an antibody drug conjugate approach. Alternatively it can also identify an antibody able to interfere with target function in downstream biological assays by the process of receptor down modulation. In this study we compare the cell surface markers identified by the proteomic and phenotypic approaches and also add functional characterisation to targets using antibodies derived from phenotypic screening.

## Results

### MDA-MB-231 membrane protein profiling using LPI™ FlowCells

The total membrane fraction from MDA-MB-231 cells was profiled and proteins were considered successfully identified if they were present in at least two flow cell replicates with at least one peptide assigned to the protein. In total, 188 proteins were successfully identified, with 37 proteins (19.7%) classified as being localised at the plasma membrane (Table 1). As expected, very few cytosolic proteins were identified and the majority of the non-plasma membrane associated proteins identified were associated with the ER/Golgi, mitochondria, cytoskeleton and nucleus. Hence, this process resulted in the identification of 37 plasma membrane proteins that are potential targets for traditional monoclonal antibody therapy. However, this identification process did not include any experimental validation of the utility of these potential targets in a disease setting and so, in parallel to this approach, two antibody-based phenotypic screens were also performed on MDA-MB-231 cells.

**Table 1:** Plasma membrane associated proteins identified from the MDA-MB-231 cell line using LPI™ FlowCells

**Protein name**	**Reproducibility (n = 5)**	**Pept. seq.**	**Seq. cov. (%)**
CD151 antigen	2	2	5.5
Guanine nucleotide-binding protein G(I)/G(S)/G(O) subunit gamma-12	5	3	34.7
Basigin	5	3	11.9
Annexin A1	5	2	5.5
CD44 antigen	5	3	5.0
Guanine nucleotide-binding protein G(s) subunit alpha isoforms XLas	5	4	4.8
Integrin a6	5	3	3.2
CD97 antigen	5	2	3.1
Integrin a3	5	2	1.8
Integrin a2	5	2	1.7
Ras-related protein Rab-35	4	3	15.4
Ephrin type-A receptor 2	4	5	5.3
Protein Shroom3	4	1	0.5
Retinoic acid-induced protein 3	3	2	5.6
MUC18	3	2	2.6
Integrin a5	3	1	1.0
Ras-related protein Rap-2b	2	2	12.0
Annexin A5	2	1	2.8
Poliovirus receptor-related protein 2	2	1	1.5
EGF receptor	2	2	1.3
Caveolin-1	5	3	15.2
Myoferlin	5	15	7.7
Myosin-9	4	3	1.5
Myosin-Ic	3	2	2.0
Plectin	5	2	3.4
HLA class I histocompatibility antigen, A-2 alpha chain	5	2	9.0
HLA class I histocompatibility antigen, B-13 alpha chain	5	2	2.5
Pyruvate kinase isozymes M1/M2	5	2	2.1
CD81 antigen	3	2	8.5
Calreticulin	5	2	11.8
Protein disulfide-isomerase	5	2	5.1
Mortalin	2	1	1.3
CD73	5	2	5.2
Monocarboxylate transporter 4	5	2	10.5
Large neutral amino acids transporter small subunit 1	5	2	6.7
Plasma membrane calcium-ransporting ATPase 1	5	2	5.2
High affinity cationic amino acid transporter 1	3	1	1.3

Proteins were identified from the SwissProt database with Mascot. The table summarizes the sequence coverage, the number of peptides sequenced and the number of runs each protein was detected (n = 5).

### Isolation of antibodies that bind and internalise in MDA-MB-231 cells using phage display

**Figure 1:**
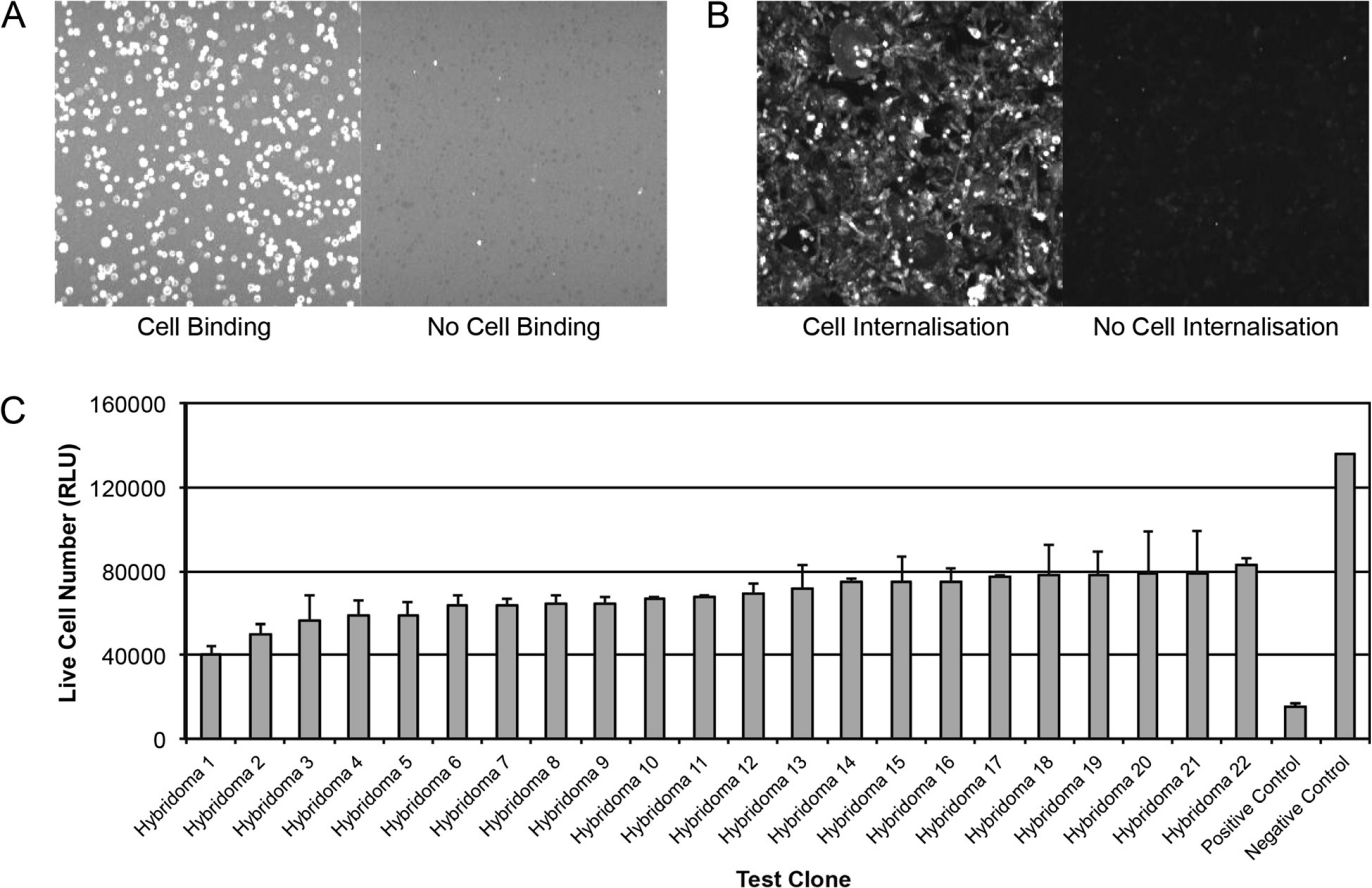
**Cell binding and internalisation screens.** (**A**) Images, from the 8200 Cellular Detection System, for cell binding and non-binding scFvs on MDA-MB-231 cells. Binding of each scFv to MDA-MB-231 cells was detected with a murine anti-HIS antibody and an Alexa647 anti-mouse antibody. (**B**) Images, from the 8200 Cellular Detection System, of an internalising scFv and a non-internalising scFv obtained from the CypHer5e internalisation screen on MDA-MB-231 cells. (**C**) Effect on MDA-MB-231 cell viability of 22 hit hybridomas able to internalise and deliver a toxic secondary antibody conjugated to saporin.

Antibody phage display was performed using MDA-MB-231 cells as the target antigen. In total, two successive rounds of cell panning were performed on these cells using a naïve single-chain variable fragment (scFv) phage library. The total output from the second round of cell panning was 2.1 × 10^5^ colony forming units (cfu) and 1,631 of these were picked and screened as soluble scFv for their ability to bind MDA-MB-231 cells and not bind ‘normal’ immortalised MCF10A cells (Figure 1A). Of the 1,631 scFv antibodies screened, 571 (35%) demonstrated binding, by fluorescent microvolume assay technology (FMAT) with an average FL1 >200, to MDA-MB-231 cells; however, 279 of these also demonstrated binding to MCF10A cells. In total, 292 scFvs demonstrated binding to MDA-MB-231 cells and had no binding to MCF10A cells. These scFvs were sequenced and a total of 52 unique scFv sequences were identified. These unique scFvs were subsequently screened for internalisation using the CypHer5E internalisation screen on MDA-MB-231 cells (Figure 1B). These scFvs were also re-screened; by flow cytometry, for cell binding specificity. Of the 52 scFvs screened, 33 (63%) were internalised, by MDA-MB-231 cells, with an average FL1 >100 (n = 3). However, upon re-screening by flow cytometry, 24 of these scFv antibodies demonstrated binding to the ‘normal’ immortalised lines MCF10A or Hs578Bst cells. Hence, 9 scFv antibodies were retained for further analysis; this is 0.6% of the starting population that had the desired cell binding specificity and cell internalisation activity.

In order to increase the number of scFv antibodies with the desired cell binding and cell internalisation activity, additional scFvs were screened. These scFv antibodies were initially isolated for their ability to bind to a wider panel of breast carcinoma cells and cell lines, including SK-BR-3, T47D, BT-474, MCF7, MDA-MB-453 and primary breast carcinoma cells, and not to MCF10A or Hs578Bst cells. Due to the practicalities of phage display, all additional cell panning experiments and screening could be performed in parallel. In total, 1007 unique scFvs were initially identified and screened for binding, by flow cytometry, to MDA-MB-231 cells and for MDA-MB-231 cell internalisation, by FMAT, using the CypHer5E internalisation screen. Antibody scFvs were retained if they showed a >10-fold MFI flow cytometry binding shift to MDA-MB-231, when compared to an isotype control, and an average FL1 >100 (n = 3) in the internalisation assay. A total of 109 additional scFvs were identified that had the desired MDA-MB-231 cell binding specificity and cell internalisation activity.

### Isolation of antibodies that bind and internalise in MDA-MB-231 cells using hybridoma technology

Mice were immunised with MDA-MB-231 cells using the repetitive immunisations at multiple sites (RIMMS) method and a total of 14,080 hybridoma supernatants were screened for their ability to bind MDA-MB-231 cells and not MCF10A cells. In parallel, the supernatants were also screened for MDA-MB-231 cell internalisation using the CypHer5E internalisation screen. In total, 4,063 (29%) supernatants demonstrated binding to MDA-MB-231 cells; however, only 1,988 (14%) supernatants bound to and were internalised by MDA-MB-231 cells and did not bind to MCF10A cells. 264 hybridoma supernatants were prioritised for further analysis and a second internalisation screen was performed that assessed the ability of the immunoglobulins (IgGs) to deliver a cytotoxic payload to MDA-MB-231 cells. The toxic payload used for this screen comprised of a secondary antibody conjugated with saporin, a toxic ribosome inactivating protein (RIP). 22 hybridoma antibodies identified as active in this cytotoxicity assay (Figure 1C), were put through clonal dilution and sequencing. Two were not carried forward and IgG was purified from 20 clonal hybridoma supernatants.

### Identification of the target antigens for the internalised antibodies

**Figure 2:**
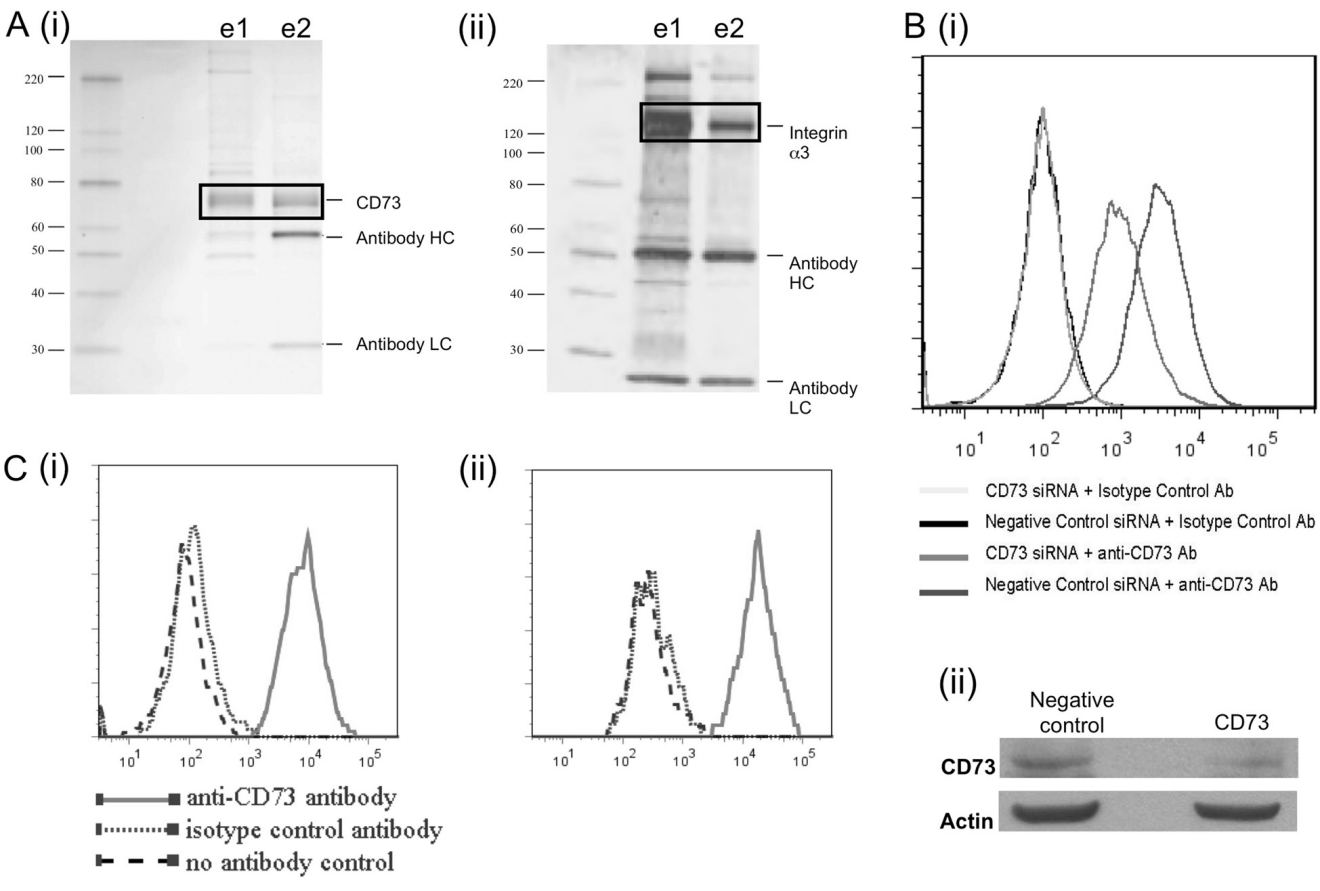
**Immunoprecipitation of target antigens and confirmation of CD73 as a target.** Immunoprecipitation (**A**) of CD73 (i) and integrin α3 (ii) from MDA-MB-231 cell lysate. MDA-MB-231 cell lysate was incubated in the presence of antibody, coupled to Sepharose. The Sepharose was washed with D-PBS prior to bound antigens being eluted using 0.1 M glycine pH 2.7 (e1 and e2). All analysis was performed by silver stained SDS-PAGE. Confirmation of target antigen for the anti-CD73 antibody was performed by siRNA knockdown of CD73 on MDA-MB-231 cells (**B**). (i) Flow cytometry was used to detect CD73 expression 48 hours post-transfection. (ii) CD73 protein knockdown in response to CD73 siRNA was determined by western blot analysis 48 hours post-transfection. Actin was used as a loading control. Binding to other triple negative breast cancer cell lines, by the anti-CD73 antibody, was confirmed by FACS (**C**). (i) Binding to SUM159 and (ii) binding to BT-549.

Target antigens for the MDA-MB-231 binding and internalising antibodies were identified by specific immunoprecipitation, peptide mass fingerprinting and tandem mass spectrometry (MS/MS) analysis. Target antigens were also identified by performing enzyme-linked immunosorbent assays (ELISAs) to recombinant forms of known surface antigens. The nine scFvs identified from phage display cell panning and screening against MDA-MB-231 cells were re-formatted as human IgG1. These antibodies, in addition to the 20 hybridoma-derived antibodies, were used to immunoprecipitate their target antigens from MDA-MB-231 lysate. Where specific proteins were immunoprecipitated the proteins were analysed by peptide mass fingerprinting and specific peptides were analysed by MS/MS. From the nine phage display-derived IgGs analysed, one (11%) immunoprecipitated a specific protein that was identified as CD73 (Figure 2A). From the 20 hybridoma-derived IgGs analysed, 16 (80%) immunoprecipitated specific proteins; seven immunoprecipitated integrin α3β1, five immunoprecipitated integrin α2β1, 3 immunoprecipitated CD44 and one immunoprecipitated galectin-3. The eight phage display-derived antibodies and the four hybridoma-derived antibodies that did not immunoprecipitate specific proteins were analysed for binding, by ELISA, to known surface antigens. From this analysis, six of the phage display-derived antibodies bound EGFR. Hence, 66% of the phage display-derived antibodies, with the desired characteristics, bound a single target. No targets were identified that were bound by antibodies isolated from both processes.

**Figure 3:**
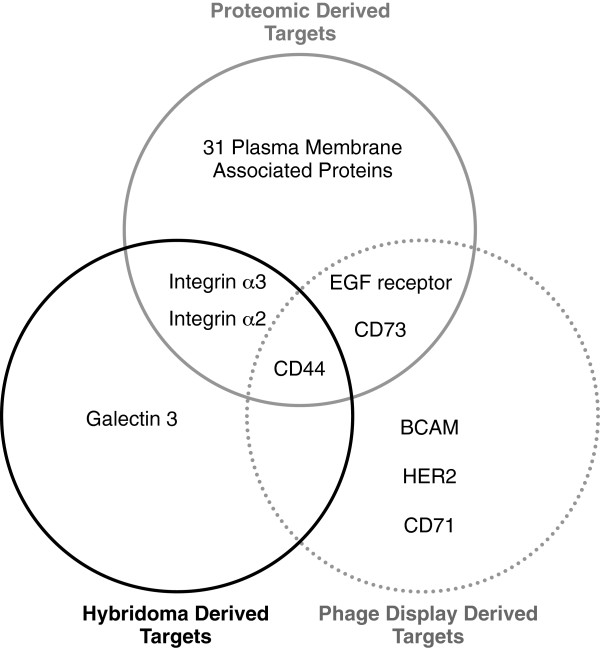
**Venn diagram summary of MDA-MB-231 plasma membrane target antigens identified by each of the three detailed target identification methods.** CD44 was identified using all three methods. Integrin α2 and integrin α3 were identified by both the hybridoma and proteomic methods. EGFR and CD73 were identified by both the phage display and proteomic methods.

The additional 109 phage display-derived scFvs, which were isolated against a wider panel of breast carcinoma cells and cell lines, including SK-BR-3, T47D, BT-474, MCF7, MDA-MB-453 and primary breast carcinoma cells, were analysed for binding, by ELISA, to known surface antigens. From this analysis, four scFvs were identified that bound BCAM, three scFvs were identified that bound HER2, two scFvs were identified that bound CD44 and 2 scFvs were identified that bound CD71. In total, nine target antigens were identified using antibody-based approaches and six of these nine were known to perform a cell communication and signal transduction role. Figure 3 compares the target antigens identified from all three approaches and only CD44 was identified by all three. Galectin-3 was unique to the hybridoma-based approach and BCAM, Her2 and CD71 were unique to the phage display-based approach but all were initially isolated against the wider panel of cell lines. 31 plasma membrane proteins were unique to the proteomic approach. Table 2 summarises and contrasts the advantages and disadvantages of using these three approaches for target discovery.

**Table 2:** Comparison of the advantages and disadvantages of membranome target discovery techniques

**Protein profiling using LPI™ FlowCells and LC-MS/MS**		**Hybridoma-based phenotypic antibody screening**		**Phage display-based phenotypic antibody screening**	
**Advantages**	**Disadvantages**	**Advantages**	**Disadvantages**	**Advantages**	**Disadvantages**
Direct comparison with normal matched tissue can be performed	Extremely reliant on quality of membrane preparation and extraction of membrane proteins	Screening antibodies can assign a potential mechanism of action	High cell requirement	Reduced cell requirement compared to hybridoma approach	Isolation of relatively low affinity antibodies and poor target identification success rate- requirement for complementary techniques for success of target identification
Sample fractionation possible and total survey of the membranome	Reliant on database annotations	Isolation of high affinity antibodies	Dominance of single targets and antibodies	Ability to perform initial screen against multiple cell types with relative ease	
	No function or mechanism of action associated with antigens identified	Functional in phenotypic screens	No ability to deselect against abundant antigens or comparator cell types	Screening can assign a potential mechanism of action	
		High target identification success rate		Ability to avoid dominance – can deselect against abundant antigens and comparator cell types	
		Isolation of antibodies that can be used for target validation or as a therapeutic candidates		Isolation of antibodies that can be used for target validation	
				Potential to identify target and therapeutic candidate	

Comparing LPI™. FlowCells and LC-MS/MS with hybridoma-based phenotypic antibody screening and phage display-based antibody screening.

### Target confirmation and validation – inhibition of MDA-MB-231 xenograft tumour growth *in vivo*

Target confirmation was achieved by performing gene knockdown experiments (Figure 2B). For the anti-CD73 antibody, derived from the phage display screen, flow cytometry analysis of cells in which CD73 gene expression had been reduced using siRNA showed uniformly reduced binding by either the test anti-CD73 antibody or a commercially available anti-CD73 antibody. This reduced binding was not observed in cells transfected with negative control siRNA. In addition to gene knockdown experiments the expression of CD73 on additional ‘triple negative’ cell lines, SUM159 and BT-549, was also confirmed by flow cytometry using the phage display-derived anti-CD73 antibody (Figure 2C).

**Figure 4:**
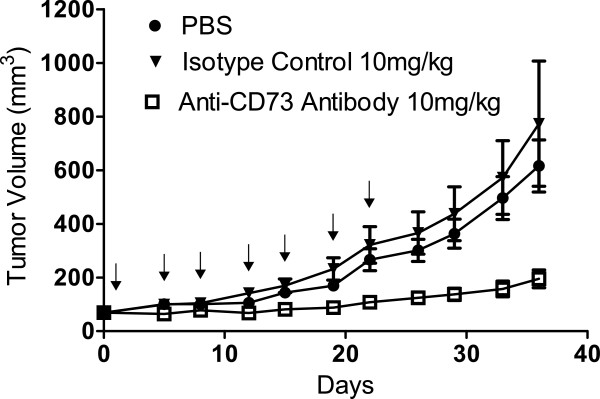
**Anti-CD73 antibody significantly inhibits the growth of pre-established MDA-MB-231 breast cancer xenograft tumours implanted into nude mice.** MDA-MB-231 cells were implanted into mice at 3x10^6^ cells/mouse and tumours were allowed to progress to 70 mm^3^. The anti-CD73 antibody or an isotype control antibody were dosed intraperitoneally on days 1, 5, 8, 12, 15, 19 and 22 (indicated by arrows). Each study group contained 10 mice.

As well as being internalised, the anti-CD73 antibody also inhibited the catalysis of adenosine monophosphate to adenosine and free phosphate (reported previously) [10]. Due to this novel mechanism and general lack of prior validation the anti-CD73 antibody was dosed in nude mice bearing MDA-MB-231 xenograft tumours to look for anti-tumour effects *in vivo*. The anti-CD73 antibody significantly reduced the growth of these tumours (Figure 4) and two weeks after the final dose of 10 mg/kg anti-CD73 IgG1, tumour size was reduced to 68% of that seen with tumours treated with a vehicle control.

## Discussion

One of the key challenges facing the development of new cancer therapies is the identification and validation of targets that are tractable to new therapies. In this study, we performed a comprehensive analysis of the plasma membranome of the MDA-MB-231 ‘triple negative’ breast carcinoma cell line with the overall aim of identifying cell surface markers tractable to antibody therapy. From a technical viewpoint, the intention was to explore the usefulness of combining a global proteomic analysis of cell surface proteins with the phenotypic screening of antibodies generated to those cell surface antigens and furthermore to compare the effectiveness of phage display and hybridoma technology for generating pools of antibodies for phenotypic screening.

Comparing the antigens identified by each method, a number were identified by multiple approaches, namely integrin-α2, integrin-α3, EGFR, CD73 and CD44, the latter being identified by all three methods. All of these antigens have been previously cited as biomarkers, potential therapeutic cancer targets or fully validated therapeutic targets, such as EGFR. Hence, this multi-approach model gives confidence to the validity of target antigens when identified by multiple approaches. Others were identified by a single method only including: BCAM, HER2, CD71, galectin-3 and the 31 antigens identified using the proteomic method. Hence, the combination of methods was effective in highlighting antibody targets relevant to tumour therapy, ranging from those already exploited in the clinic to those which could be of great interest as novel antibody targets. For this purpose, the benefit of using a phenotypic screening approach, which provides an antibody reagent for further validation work and potentially a therapeutic lead, was clearly illustrated by the use of the phage display-derived anti-CD73 antibody to inhibit MDA-MB-231 tumour growth *in vivo*, as discussed later.

Looking in more detail at each method and first considering the proteomic approach, it was shown that the global analysis of membrane protein content identified 37 plasma membrane proteins. A further 74 proteins were also believed to be derived from internal membranes such as those from ER/golgi/ribosome, mitochondria, and nucleus. In order to assign these proteins to their cellular location, we were reliant on prior classifications in public databases with varying levels of experimental validation and therefore may have overlooked the possibility that some proteins had relocated to the plasma membrane as part of the oncogenic process. Nevertheless, this list of 37 plasma membrane proteins provided a useful survey of the membrane protein content and contained some well validated targets, such as EGFR. It also included others that were not identified by the phenotypic antibody screening approaches that have been implicated in cancer progression, metastasis and invasion including: CD151 antigen, basigin, ephrin type-A receptor 2 and myoferlin. However, it does contain a large number of targets for which disease linkage is not well established. As such, these remaining targets would require significant experimental work to validate their utility for antibody intervention. The total number of membrane proteins identified was also relatively low, bearing in mind the total estimated number of human membrane proteins present [11]; however, this may reflect the processing steps required to enrich for pure membrane fractions.

An alternative approach to focus on membrane proteins upregulated in cancerous cells would have required a comparative analysis of matched, normal cell lines. However, it can be extremely challenging to procure sufficient quantities of well-matched normal material for such studies and this would ultimately not lead to functional validation of targets but would remove those targets that are common between the two cell types. Further optimisation of the proteomic method presented here may also be beneficial. This type of ‘shot-gun’ proteomics method often leads to a highly complex mixture of peptides at varying concentrations, thus placing high demands on the liquid chromatography-tandem mass spectrometry (LC-MS/MS) step for identifying the peptides. Longer separation times, or the use of two-dimensional LC, in which the sample is first divided into fractions using a strong cation exchange column, followed by standard reverse phase separation, could potentially enable the discovery of additional targets amenable to antibody therapy.

To explore phenotypic screening approaches for target identification, both phage display and hybridoma technologies were investigated in this study. Both approaches used two main criteria for prioritising antibodies tailored to antibody characteristics we were interested in. These were (i) preferential binding to MDA-MB-231 cells over normal, immortalised breast cell lines, and (ii) internalisation in MDA-MB-231 cells. Importantly, both the binding and internalisation screens could be performed in high-throughput in order to assay thousands of antibodies for function. Using the hybridoma method, we observed that four target antigens could be identified; namely integrin-α2, integrin-α3, CD44 and galectin-3. The last of these was not a true plasma membrane protein but rather a secreted protein which can associate with the cell membrane as a result of its carbohydrate recognition domains [12]. One of the advantages of the hybridoma approach was that the antibodies were highly effective immunoprecipitation reagents, with 16 of the final 20 hybridoma antibodies able to immunoprecipitate a specific target, presumably on the basis that they have undergone affinity maturation *in vivo*. This high affinity also gives the potential that the antibodies could be directly used as a therapeutic, without further optimisation, if relevant criteria are fulfilled. However, this affinity maturation could also be a disadvantage because several of the antibodies in the final 20 were minor sequence variants of each other. This limited the number of target antigens identified and any future immunisation process would require optimisation to avoid such clonal dominance, either by extensive sequencing early on in the screening process or by shortening the immunisation procedure.

Using the phage display approach on the MDA-MB-231 cell line, the initial screen was able to isolate nine different scFv antibodies that fulfilled the screening criteria. However, due to the speed and ease with which further selections and screening could be performed, we were able to identify an additional 109 functional scFvs from other cell lines. In total, this approach identified six targets: EGFR, B-CAM, CD44, CD73, CD71 and HER2. All of these have previously been implicated in cancer progression, with the least validation associated with CD73 [13,14]. It must be noted that the MDA-MB-231 cell line is described as HER2 negative, and it does not amplify or overexpress HER2. However, it does have a basal level of HER2 expression [15] that could be sufficient to illicit a functional response. Hence, direct cell panning on MDA-MB-231 cells failed to identify anti-HER2 antibodies; however, when antibodies were screened, which had been isolated against other cell types; internalising anti-HER2 antibodies were identified. This highlights the advantages of not restricting a screen to those antibodies that have only been isolated against the test cell type.

The success rate of immunoprecipitation using the phage-display antibodies was low, with only 1 of the final 9 antibodies able to immunoprecipitate a specific target, CD73. This is most probably due to the relatively low affinity as these antibodies were isolated from a naïve library. Target identification success rate was increased by performing ELISAs against known targets and could be improved further by incorporating cDNA-based target identification methods. However, even without affinity maturation and further development this study also demonstrates that an antibody derived from this phage display-based approach can be used to validate an identified target. The anti-CD73 antibody isolated by phage display demonstrated anti-tumour activity in a MDA-MB-231 xenograft model and highlights the advantage that isolating a target and an antibody together can accelerate the early validation of that target in a disease relevant setting. This can be illustrated further by using CD73 as an example. In 1991, Kruger et al. demonstrated expression of CD73 in breast carcinoma [16] and a further 19 years of target biology exploration and experimental validation ensued before Stagg et al. demonstrated that an anti-CD73 antibody could inhibit breast tumour growth and metastasis [17]. This also required the *de novo* generation of antibody tool reagents in order to test the inhibition hypothesis in a disease model. In the phenotypic screening approach described here, in which antibody generation was an integral part, the whole process from initial screen to *in vivo* target validation took approximately 12 months. In addition to this *in vivo* validation these antibodies can also be used to establish disease association via immunohistochemistry of patient tissue samples and in mechanistic studies to understand the optimal mode of action.

This study focused on a single ‘triple negative’ breast cancer cell type. Focusing on a single cell type has two implications. The first is that this kind of analysis is ideal for a personalised healthcare approach if a suitable target cell type can be identified and isolated. Here were have focused on a ‘triple negative’ breast cancer cell type; however, this could easily be substituted for another cell type such as a KRAS-mutant non small cell lung carcinoma cell type. The second implication is the potential to identify many more targets by looking at other key disease-promoting cell types and not just restricting this approach to tumour cell types.

## Conclusions

This study has demonstrated that multiple methods are required to successfully interrogate the membranome of a desired cell type, with a total of 40 target antigens identified for the MDA-MB-231 cell type. It has also successfully demonstrated that phenotypic antibody screening provides a mechanism for rapidly discovering and evaluating antibody tractable targets, which can significantly accelerate the therapeutic discovery process. Two phenotypic antibody screening approaches were evaluated with targets being identified by both. The hybridoma-based method identified antibodies capable of immunoprecipitating their target antigen, however, these antibodies lacked diversity. The phage display-based method identified antibodies that did not perform well in immunoprecipitation experiments; however targets were identified and one of these antibodies, against CD73, was able to demonstrate anti-tumour activity *in vivo*. By evaluating *in vivo* function early in the discovery process, the suitability of a target for therapeutic intervention can be assessed at the very beginning of the discovery process, lowering the risk of attrition further downstream and increasing the likelihood of success.

## Methods

### Cell culture

MDA-MB-231 (NCI, Bethesda, MD), MCF10A, Hs578Bst, T47D, BT474, SKBR3, MDA-MB-453, BT-549 (ATCC, Manassas, VA), SUM159 and primary breast cancer (Asterand, Detroit, MI) cells were cultured according to supplier’s guidelines.

### Membrane protein profiling using LPI™ FlowCells and LC-MS/MS

Membrane protein profiling was performed essentially as described by Padliya *et al*., 2009 [18]. Briefly, MDA-MB-231 cells were subjected to membrane preparations whereby membrane proteins from all membrane types were isolated. The cells were diluted with NaHCO_3_ buffer and lysed with a Dounce homogenizer. Unbroken cells and nuclei were removed by centrifugation and the membranes in the post-nuclear supernatant were treated with Na_2_CO_3_, pH 11 to remove membrane-associated proteins. The final membrane preparation was suspended in Tris-NaCl buffer and treated with a VibraCell™ sonicator (Model 501, Sonics&Materials, Inc.) equipped with a 2 mm microtip to prepare proteoliposomes. The prepared proteoliposomes were immobilized onto the surface of the LPI™ FlowCell and subjected to wash protocols prior to the addition of Trypsin for the preparation of peptides. The peptides were eluted from the LPI™ FlowCell and post-digested overnight before each peptide sample was subjected to reverse phase (RP) LC-MS/MS analysis. Peptides were separated using a 40 min gradient on a 180 mm RP column (50 μm i.d.) with a flow of 100 nm/min. Mass analysis was performed using a 7-Tesla LTQ-FT Mass Spectrometer (Thermo Electron, Waltham, Massachusetts). Raw MS data from each sample was collected, processed and searched using the MASCOT algorithm.

### Phage display antibody isolation

Phage display cell panning was performed to isolate scFv antibody fragments. A large naïve human scFv phage display library, containing up to 1×10^11^ binding members [19] was used for antibody isolation as described previously [20]. Briefly, the phage library and 1×10^7^ cells were individually blocked for 1 hour at room temperature in phosphate-buffered saline (PBS) containing 3% (w/v) Marvel milk powder. Blocked phage were added to the cells and incubated for 1 hour at room temperature. The supernatant was then removed and the cell pellet was washed 6 times with PBS. The phage were then recovered using triethanolamine cell treatment and amplified in *E. coli* as described previously [20]. Following phage display cell panning, individual scFvs were expressed into the culture supernatant and screened for cell binding using the 8200 Cellular Detection System (Applied Biosystems, Carlsbad, CA). Periplasmic extracts, containing each scfv, were also prepared and used to screen for scFv internalisation via detection of CypHer5E (GE Healthcare, Bucks, UK) fluorescence on the 8200 Cellular Detection System (Applied Biosystems, Carlsbad, CA). Periplasmic extracts were also used to provide the scFvs for cell binding analysis using flow cytometry.

### Hybridoma antibody isolation

Mice were immunised with MDA-MB-231 cells using a RIMMS protocol [21]. All work was undertaken under the 1986 Animals (Scientific Procedures) act in fully Home Office licensed facilities under an approved Home Office Project Licence. For immunised mice, the terminal bleed occurred at day 28 and serum ELISAs were performed on all bleeds to determine the immune response to MDA-MB-231 cells. The mice with the greatest immune response to MDA-MB-231 cells were selected for fusions. Fusions were performed essentially as described by Kohler & Milstein, 1975 [22]. Hybridoma supernatants were screened for cell binding and internalisation using the 8200 Cellular Detection System (Applied Biosystems, Carlsbad, CA). Antibodies were purified from a subset of hybridoma supernatants using PhyTip® columns containing Protein A affinity resin (PhyNexus, Inc, San Jose, CA), according to the manufacturer’s instructions. Purified antibodies were incubated with the Mab-ZAP reagent (Advanced Targeting Systems, San Diego, CA) and MDA-MB-231 cells for 72 hours at 37°C prior to evaluating cell viability using a CellTitre-Glo® luminescent viability assay (Promega, Madison, WI) according to the manufacturer’s instructions.

### Antibody target identification - immunoprecipitation and MS/MS

Prior to antibody target identification, phage display-derived scFvs were reformatted as human IgG1. Briefly, the V_H_ and V_L_ from each scFv were sub-cloned into appropriate heavy chain (HC) and light chain (LC) mammalian transient expression vectors and expressed in Chinese Hamster Ovary (CHO) cells. Recombinant IgG1 were purified from the CHO cell culture supernatant using MabSelect SuRe™ affinity columns (GE Healthcare, Bucks, UK), according to the manufacturer’s instructions. The same purification method was also used to purify antibody from the clonal hybridoma cell culture supernatants. For each immunoprecipitation, 1×10^8^ MDA-MB-231 cells were lysed in 30 ml PBS containing 1% Triton X-100 (w/v) and 1× protease inhibitors (Roche Applied Science, West Sussex, UK). A total of 1 mg of purified antibody was coupled to CNBr-activated Sepharose™ 4B according to the manufacturer’s specifications. The cell lysate was incubated in the presence of the antibody coupled to Sepharose before the antigens bound to the Sepharose were eluted with 0.1 M glycine, pH 2.7. Eluted fractions were analysed on a silver-stained sodium dodecyl sulfate polyacrylamide gel electrophoresis (SDS-PAGE) gel (Invitrogen, Paisley, UK). Bands of protein present in the eluted fractions were excised from the gel, subject to in-gel digestion and the protein identified through a combined approach of peptide mass fingerprint searches using MASCOT ( http://www.matrixscience.com) and MS/MS sequencing by matrix-assisted laser desorption/ionisation time-of-flight (MALDI-TOF) MS (TOPlab, Martinsried, Germany).

### Antibody target identification - recombinant protein ELISA

ScFv periplasmic samples (from phage display panning) and murine antibodies (from hybridoma immunisations) were screened for binding to known cell membrane proteins by ELISA. Briefly, recombinant proteins (R&D Systems, Abingdon, UK) were plated overnight before incubation with either scFv or murine IgG samples. Detection was achieved using an appropriate horseradish peroxidise-conjugated secondary antibody and tetramethylbenzidine as a substrate (Pierce, Rockford, IL).

### Antibody target confirmation and validation in a MDA-MB-231 xenograft

Prior to *in vivo* study the target for the anti-CD73 antibody was confirmed by siRNA knockdown. Briefly, MDA-MB-231 cells were reverse transfected using RNAiMAX, according to the manufacturer’s instructions (Invitrogen, Paisley, UK), and 50 nM siRNA (Dharmacon, Lafayette, CO). After 48 hours, cell surface expression of CD73 was determined, by FACS, using the phage display-derived anti-CD73 IgG1 and a commercial anti-human CD73 antibody (BD Pharmingen, Franklin Lakes, NJ). Cells were also lysed and analyzed by western blot. CD73 expression was detected using a commercial anti-human CD73 antibody (Santa Cruz Biotechnology Inc, Santa Cruz, CA).

For *in vivo* analysis, MDA-MB-231 cells were implanted subcutaneously into the right flank of female, athymic nude mice (5–7 weeks of age) at 3×10^6^ cells/mouse and tumours were allowed to progress to 70 mm^3^. Mice were purchased from Harlan, (Indianapolis, IN) and there were 10 mice per study group. Antibodies were dosed intraperitoneally twice weekly at 10 mg/kg until day 22. Tumours were measured twice weekly in two dimensions with calipers and tumour volume was calculated as [L(length) × W^2^(width)]/2. Animals were housed in a USDA registered, AAALAC accredited animal facility in accordance with the Guide for Care and Use of Laboratory Animals. All experiments were performed in compliance with the institutional animal care guidelines under an IACUC-approved animal protocol.

## Abbreviations

LC-MS/MS: Liquid chromatography-tandem mass spectrometry; scFv: Single-chain variable fragment; FMAT: Fluorescent microvolume assay technology; RIP: Ribosome inactivating protein; RIMMS: Repetitive immunisations at multiple sites; ELISA: Enzyme-linked immunosorbent assay; PBS: Phosphate-buffered saline; HC: Heavy chain; LC: Light chain; SDS-PAGE: Sodium dodecyl sulphate-polyacrylamide gel electrophoresis; AAALAC: Association for Assessment and Accreditation of Laboratory Animal Care; USDA: United States Department of Agriculture; IACUC: Institutional Animal Care and Use Committee.

## Competing interests

The authors declare no competing financial interests.

## Authors’ contributions

SR and SG designed the experiments and performed the phage display antibody isolation. JE planned and coordinated the mouse immunisations and the hybridoma antibody isolation. MD, AK and RK performed the membrane protein profiling using LPI™ FlowCells and LC-MS/MS. KS and EB designed and performed the internalisation assays. DL, GK, CH and MF performed antibody target identification, confirmation and validation. RH, LJ and RM conceived of the study, and participated in its design and coordination. SR wrote the manuscript with comments from all authors. All authors read and approved the final version of the manuscript.
